# P2X7R promotes angiogenesis and tumour‐associated macrophage recruitment by regulating the NF‐κB signalling pathway in colorectal cancer cells

**DOI:** 10.1111/jcmm.15708

**Published:** 2020-07-31

**Authors:** Chunhui Yang, Shuang Shi, Ying Su, Jing‐Shan Tong, Liangjun Li

**Affiliations:** ^1^ Department of Clinical Laboratory the Second Affiliated Hospital of Dalian Medical University Dalian China; ^2^ Department of Pharmacology and Chemical Biology University of Pittsburgh School of Medicine Pittsburgh PA USA

**Keywords:** angiogenesis, cancer stem cells, NF‐κB, P2X7R, TAMs, tumour growth

## Abstract

Overexpression of P2X7R has been observed in several tumours and is related to cancer advancement and metastasis. However, the role of P2X7R in colorectal cancer (CRC) patients is not well understood. In the current study, overexpression of P2X7R and the effects at the molecular and functional levels in CRC were assessed in a mouse orthotopic model. Functional assays, such as the CCK‐8 assay, wound healing and transwell assay, were used to determine the biological role of P2X7R in CRC cells. CSC‐related genes and properties were detected via sphere formation and real‐time PCR assays. The underlying mechanisms were explored by Western blotting, real‐time PCR and Flow cytometry. In this study, we found that overexpression of P2X7R increases in the in vivo growth of tumours. P2X7R overexpression also increased CD31, VEGF and concurrent angiogenesis. P2X7R up‐regulates aldehyde dehydrogenase‐1 (ALDH1) and CSC characteristics. Transplanted tumour cells with P2X7R overexpression stimulated cytokines to recruit tumour‐associated macrophage (TAMs) to increase the growth of tumours. We also found that the NF‐κB signalling pathway is involved in P2X7R‐induced cytokine up‐regulation. P2X7R promotes NF‐κB–dependent cytokine induction, which leads to TAM recruitment to control tumour growth and advancement and remodelling of the stroma. Our findings demonstrate that P2X7R plays a key role in TAM recruitment, which may be a therapeutic target for CRC patients.

## 1 BACKGROUND

The number of deaths due to colorectal cancer (CRC) stands at 0.7 million with diagnosis of 1.4 million new cases across the globe.^1^, ^2^ The heterogeneous nature of this disease suggests the involvement of genetics such as chromosomal and microsatellite instability and epigenetics such as the CpG island methylation phenotypes.^3^, ^4^, ^5^

Angiogenesis and the ability to metastasize involve tumour‐associated macrophages (TAMs) in CRC.^6^, ^7^ M1 and M2 are the major classes of TAMs that display polar effects on tumours.^8^, ^9^ The former class is activated classically by lipopolysaccharide (LPS) and interferon‐γ (IFN‐γ) to secrete interleukin‐12 (IL‐12), IL‐1β and cytotoxic inducible nitric oxide synthase (iNOS), among other factors.^10^, ^11^ Alternative activation is observed in M2 macrophages due to IL‐4, IL‐10 or IL‐13, which express vascular endothelial growth factor (VEGF) and other factors for angiogenesis, as well as IL‐6 and IL‐10.^12^, ^13^ The infiltration of increased numbers of M2 macrophages is associated with poor prognosis in patients with colon cancer.^6^, ^14^ TAM polarization and differentiation is controlled by the microenvironment and tumour cells themselves by means of cytokines synthesized by the latter.^15^, ^16^

Lymphocytes express P2X7R (P2X purinoceptor 7) to control activation of T cells (mainly T helper 1 [Th1] and Th17 cells) via extracellular adenosine 5′‐triphosphate (ATP) secreted by damaged cells.^17^, ^18^ These T cells function in alloimmune responses as well as the rejection of allografts.^19^ The receptor, which exists in several single nucleotide polymorphisms (SNPs) and splice isoforms (A–J), correlates with many diseases. The P2X7R is a ligand‐gated ion channel permeable to Ca^2+^, K^+^ and Na^+^.^20^ Prolonged stimulation of this receptor leads to pore formation that causes the entry of large molecules, resulting in cell lysis and mortality.^21^ Several of the functions of P2X7R in cancer include inducing cell death and serving as an antitumour receptor.^21^, ^22^ Another role involves in sustaining the and the ability of cancer cells to migrate and invade in vitro and in vivo.^23^ These apparently contradictory roles can be explained by the involvement of isoforms: P2X7A for apoptosis and P2X7B for stimulation.^24^ Because the involvement of P2X7R in modulating the immune system and inflammatory pathways is known, its role in tumour biology is of interest, especially because this receptor is overexpressed in several tumours and is linked to reduced survival and increased advancement and metastasis.

This work is an investigation of the involvement of P2X7R in the function of CRC cells. The observations show an increase in cancer stem cells (CSCs), VEGF up‐regulation to cause angiogenesis and increased growth of tumours in vivo. Overexpression of P2X7R caused TAM recruitment via the involvement of high levels of VEGF, (C‐C motif) ligand 2/5 (CCL2/5) and macrophage‐specific colony‐stimulating factor (CSF‐1).

## 2 MATERIALS AND METHODS

### 2.1 Cell culture

Human CRC cell lines, including HCT116, DLD1, SW837, and RKO and the BALB/C‐derived mouse colon adenocarcinoma cell line CT26, were obtained from ATCC (American Type Culture Collection, Manassas, VA, USA). All cell lines were cultured in Dulbecco's modified Eagle's medium (DMEM) with 4.5 g/L glucose (Mediatech, Manassas, VA, USA) supplemented with 10% FBS at 37°C in a humidified atmosphere of 5% CO_2_.

### 2.2 P2X7R overexpression and knockdown

Generation of pcDNA3.1‐hP2X7R, an expression plasmid for P2X7R, was performed as previously described.^25^ Lipofectamine 3000 was used to transfect RKO and HCT116 cells using the control pcDNA3.1 or pcDNA3.1‐hP2X7R. pcDNA3.1‐mP2X7R was constructed by cloning murine P2X7R cDNA into the EcoRI and NotI sites of pcDNA3.1. This was followed by the stable transfection of CT26 cells using control pcDNA3.1 or pcDNA3.1‐mP2X7R and Lipofectamine 3000 (Invitrogen, Waltham, MA, USA) according to the prescribed protocols. Zeocin (300 µg/mL; Invitrogen, Waltham, MA, USA) in the medium of CT26‐Con and CT26‐mP2X7R cells was used to select stable transfectants.

Lipofectamine 3000 was utilized for transfection of HEK293T cells with green fluorescent protein (GFP)‐tagged lentiviral (pGIPZ) constructs containing P2X7R shRNA (sh*P2X7R*, 5′‐GGAUCCAGAGCAUGAAUUAUU‐3′) or scrambled shRNA (shCon; Dharmacon, Lafayette, CO, USA) and pCMV‐∆8.2 and pCMV‐VSVG packaging plasmids (Addgene, Watertown, MA, USA). At 72 hours after transfection, viral supernatants were collected, followed by instant infection in the presence of 10 μg/mL polybrene (Sigma‐Aldrich, St. Louis, MO, USA). Puromycin at a concentration of 5 μg/mL was utilized for the selection of the above‐mentioned transfectants, which were subjected to cell sorting, and the top 10%‐20% cells with staining for GFP were collected.

### 2.3 Animal models

The Second Affiliated Hospital of Dalian Medical University Institutional Animal Care and Use Committee guidelines issued approval for the experiments. The establishment of the orthotopic CRC mouse model was in accordance with a previously described protocol.^26^ Briefly, 2% isoflurane in oxygen was administered to BALB/C mice (8 weeks of age) by inhalation for anaesthesia. The caecum was exposed by making a midline incision. A 33‐gauge microinjector (Hamilton) was used to inject 10 μL of 2 × 10^6^ CT26‐Con or CT26‐mP2X7R cells in phosphate buffered saline (PBS) into the subserosa of the caecum. Tissue adhesive (3 M) was used to seal the site to avoid leakage, followed by washing with 70% alcohol and PBS. Then, 6‐0 polyglycolic acid sutures (CP Medical, Norcross, GA, USA) were used for skin and abdominal wall closure following the replacement of the caecum in the peritoneal cavity. The sacrifice of the animals was performed 6 weeks after the cell implantation, followed by measuring the tumour weights and processing the tumours.

### 2.4 Histology and immunohistochemistry

The processing of tumour samples was performed in accordance with previously established protocols.^27^, ^28^ Normal horse serum (Jackson ImmunoResearch, West Grove, PA, USA) was applied for 60 minutes to block non‐specific epitopes. This was followed by incubation of the sections at 4°C overnight with primary antibodies. For immunohistochemistry, the 2‐Solution diaminobenzidine (DAB) kit (Invitrogen, Waltham, MA, USA) was utilized to detect signals following a 60 minutes incubation with suitable secondary antibodies conjugated to HRP (Bio‐Rad, Hercules, CA, USA) at room temperature. This was followed by haematoxylin counterstaining and mounting in Acrymount (StatLab). The staining was evaluated at ×400 magnification in a minimum of three areas and based on the scores, were categorized per specimen as follows: score 0, negative; score 1, <25% positive cells; score 2, 25%‐50% positive cells; score 3, 50%‐75% positive cells; and score 4, >75% positive cells. This was followed by scoring of immunostaining intensity as follows: 1+, weak; 2+, moderate; and 3+, intense. A total score was obtained by multiplying the two above‐mentioned values. Two experienced investigators blinded to the experimental groups and to each other evaluated the immunostaining independently.

### 2.5 ELISA

Cytokine quantification levels of CSF‐1 (Thermo Fisher Scientific, EHCSF1 for human and EMCSF1 for mouse, respectively), CCL2 (Thermo Fisher Scientific, Waltham, MA, USA, BMS281 for human and BMS6005 for mouse, respectively) and CCL5 (Thermo Fisher Scientific, Waltham, MA, USA, EHRNTS for human and KMC1031 for mouse, respectively) secreted in the conditioned media (24 hours after transfection) were quantitated by ELISA according to the manufacturer's instructions.

### 2.6 Cell viability assay

A total of 3000 human or mouse CRC cells with or without P2X7R transfection in 100 μL of complete culture media per well were added to 96‐well plates. For NF‐κB inhibition, P2X7R overexpression cells were treated with CAPE or JSH‐23. CCK‐8 (Sigma‐Aldrich, St. Louis, MO, USA) reagent was utilized to assess proliferation at 72 hours after plating in accordance with the manufacturer's protocols.

### 2.7 Western blotting

Western blotting was performed as previously described.^26^ Briefly, RIPA buffer (Pierce, Rockford, IL, USA) was utilized to prepare whole cell lysates as previously described. Centrifugation of the final lysate was performed at 13 000 *g* for 15 minutes at 4°C. Reduction of the samples was performed by heating for 10 minutes with 50 mmol/L DTT at 98°C. Ten per cent polyacrylamide precast gels (Invitrogen, Waltham, MA, USA) were used to separate 50 μg of protein, followed by blotting to PVDF membranes (Invitrogen, Waltham, MA, USA). This was followed by blocking the membranes with a 5% skim milk solution in TBS‐Tween 0.05% buffer at room temperature for 60 minutes. Next, the membranes were incubated overnight with primary antibodies at 4°C. Secondary antibodies conjugated to HRP were incubated with the blots, followed by development using EZ‐ECL (Biological Industries, Cromwell, CT, USA) and visualization on a Fusion FX (Vilber Lourmat, Collégien, France). The primary antibodies used in this study are list as follows: P2X7R (APR‐004, Alomone Labs, Jerusalem, Israel), E‐cadherin (ab40772, Abcam, Cambridge, MA, USA), N‐cadherin (ab76011, Abcam, Cambridge, MA, USA), Vimentin (ab8978, Abcam, Cambridge, MA, USA), VEGF (ab32152, Abcam, Cambridge, MA, USA), MMP7 (ab207299, Abcam, Cambridge, MA, USA), MMP9 (ab38898, Abcam, Cambridge, MA, USA), KLF4 (ab214666, Abcam, Cambridge, MA, USA), ALDH1 (36671, Cell Signaling Technology, Danvers, MA, USA), ABCG2 (4477, Cell Signaling Technology, Danvers, MA, USA), Nanog (8822, Cell Signaling Technology, Danvers, MA, USA), c‐Myc (9402, Cell Signaling Technology, Danvers, MA, USA), SOX2 (3579, Cell Signaling Technology, Danvers, MA, USA), CCL2 (2027, Cell Signaling Technology, Danvers, MA, USA), CCL5 (2987, Cell Signaling Technology, Danvers, MA, USA), CSF‐1 (3155, Cell Signaling Technology, Danvers, MA, USA), HIF‐1α (36169, Cell Signaling Technology, Danvers, MA, USA), HIF‐1β (3414, Cell Signaling Technology, Danvers, MA, USA), β‐actin (3700, Cell Signaling Technology, Danvers, MA, USA), HO‐1 (82206, Cell Signaling Technology, Danvers, MA, USA) and c‐MET (8041, Cell Signaling Technology, Danvers, MA, USA).

### 2.8 Flow cytometry analysis

Anti‐CD44 conjugated to FITC and anti‐CD166 conjugated to PE were used for staining CT26‐Con and CT26‐mP2X7R cells. Sequential enzymatic digestion was utilized to obtain single‐cell suspensions from tumours as previously described.^29^ A 70 µm nylon cell strainer (BD Biosciences, Franklin Lakes, NJ, USA) was utilized to filter the suspensions, followed by double PBS washing. Staining of the isolated CT26‐Con or CT26‐mP2X7R cells was performed using anti‐Gr1‐FITC, CD11b‐APC and anti‐F4/80‐PE (eBioscience, San Diego, CA, USA). A total of 1 × 10^6^ cells was incubated with antibodies in 100 μL wash buffer (PBS plus 0.1% BSA) for 60 minutes at 4°C. After washing with the same cold buffer, 1% paraformaldehyde was utilized for fixation. A BD LSRII flow cytometer (BD Biosciences, Franklin Lakes, NJ, USA) was utilized for flow cytometric analysis of 30 000 cells.

### 2.9 In vitro formation of tumorspheres

Ultralow attachment 6‐well plates (Corning, NY, USA) were used to plate CT26‐Con, CT26‐mP2X7R, HCT116‐Con, HCT116‐hP2X7R, HCT116‐sh con and HCT116‐sh hP2X7R cells (10 000 per well) in 2 mL of serum‐free medium in accordance with established protocols. After 1 week of culture at 37°C and 5% CO_2_, manual counting of the tumourspheres was performed for each well.

### 2.10 Assessing wound healing, migratory and invasive abilities

CT26‐Con and CT26‐mP2X7R cells were seeded in six‐well plates and cultured until reaching 90% confluence. A sterile 200 µL pipette tip was utilized to wound the monolayers, followed by incubation in DMEM plus 1% FBS for 24 hours under standard conditions. Marking and photography of the wounded areas before and after incubation was performed. ImageJ (NIH) was utilized to measure the area of the wound to assess the repair in triplicate assays and triplicate wells. The ability of the P2X7R‐overexpressing cells and appropriate controls and P2X7R‐knockdown cells and their appropriate controls to invade and migrate was performed in accordance with a previous protocol in triplicate across triplicate wells.

### 2.11 Real‐time PCR

Real‐time PCR was performed as previously described.^30^ Briefly, a Total RNA Isolation Mini Kit (Agilent, Santa Clara, CA, USA) was utilized to isolate the total RNA from cells. The RevertAid First Strand cDNA synthesis kit (Fermentas, Waltham, MA, USA) was used to synthesize complementary DNA. The RT‐PCR cDNA involved the use of primers, Jumpstart Taq DNA and a dNTP mix. The parameters included one cycle for 1 minutes at 95°C, 45 cycles for 20 seconds at 95°C, 30 seconds at 57°C, and 1 minutes at 72°C, and one final cycle for 5 minutes at 72°C. Roche FastStart Universal SYBR Green Master (ROX) was utilized for the real‐time PCRs using the following parameters: one cycle for 2 minutes at 50°C and one cycle for 10 minutes at 95°C. Subsequently, 35 amplification cycles of 15 seconds at 95°C, 60 seconds at 57°C and 60 seconds at 72°C were performed. The final step was a dissociation step for 15 seconds at 95°C and 15 seconds each at 60°C and 95°C. The primers are list as follows: Nanog, F/R: 5′‐AGGGTCTGCTACTGAGATGCTCTG‐3′/5′‐CAACCACTGGTTTTTCTGCCACCG‐3′; KLF4, F/R: 5′‐GCAATATAAGCATAAAAGATCACCT‐3′/5′‐AACCAAGACTCACCAAGCACC‐3′; ALDH1, F/R: 5′‐CTGCTGGCGACAATGGAGT‐3′/5′‐GTCAGCCCAACCTGCACAG‐3′; c‐Myc, F/R: 5′‐TGAGGAGACACCGCCCAC‐3′/5′‐CAACATCGATTTCTTCCTCATCTTC‐3′; ABCG2, F/R: 5′‐CCATAGCCACAGGCCAAAGT‐3′/5′‐GGGCCACATGATTCTTCCAC‐3′; SOX2, F/R: 5′‐GGCAGCTACAGCATGATGCAGGAGC‐3′/5′‐CTGGTCATGGAGTTGTACTGCAGG‐3′; CSF‐1, F/R: 5′‐CATCTCAGCCCCACCTGCATGGTA‐3′/5′‐TCCTGGGCAGGAAGGAAAGTC‐3′; CCL2, F/R: 5′‐AGGTCCCTGTCATGCTTCTG‐3′/5′‐TCTGGACCCATTCCTTCTTG‐3′; CCL5, F/R: 5′‐ACTCCCTGCTGCTTTGCCTAC‐3′/5′‐GAGGTTCCTTCGAGTGACA‐3′; GAPDH, F/R: 5′‐GCACAGTCAAGGCCGAGAAT‐3′/5′‐GCCTTCTCCATGGTGGTGAA‐3′.

### 2.12 Statistical analysis

The data are expressed as the mean ± standard deviation (SD). The comparison of 2 sets was performed utilizing Student's *t* test, while one‐way ANOVA followed by Dunnett's test was utilized for more than 2 data samples. Significance was considered at *P* < 0.05, *P* < 0.01 or *P* < 0.001, which are represented by *, ** or ***, respectively.

## 3 RESULTS

### 3.1 P2X7R promotes CRC cell proliferation, migration and invasion in vitro

**FIGURE 1:**
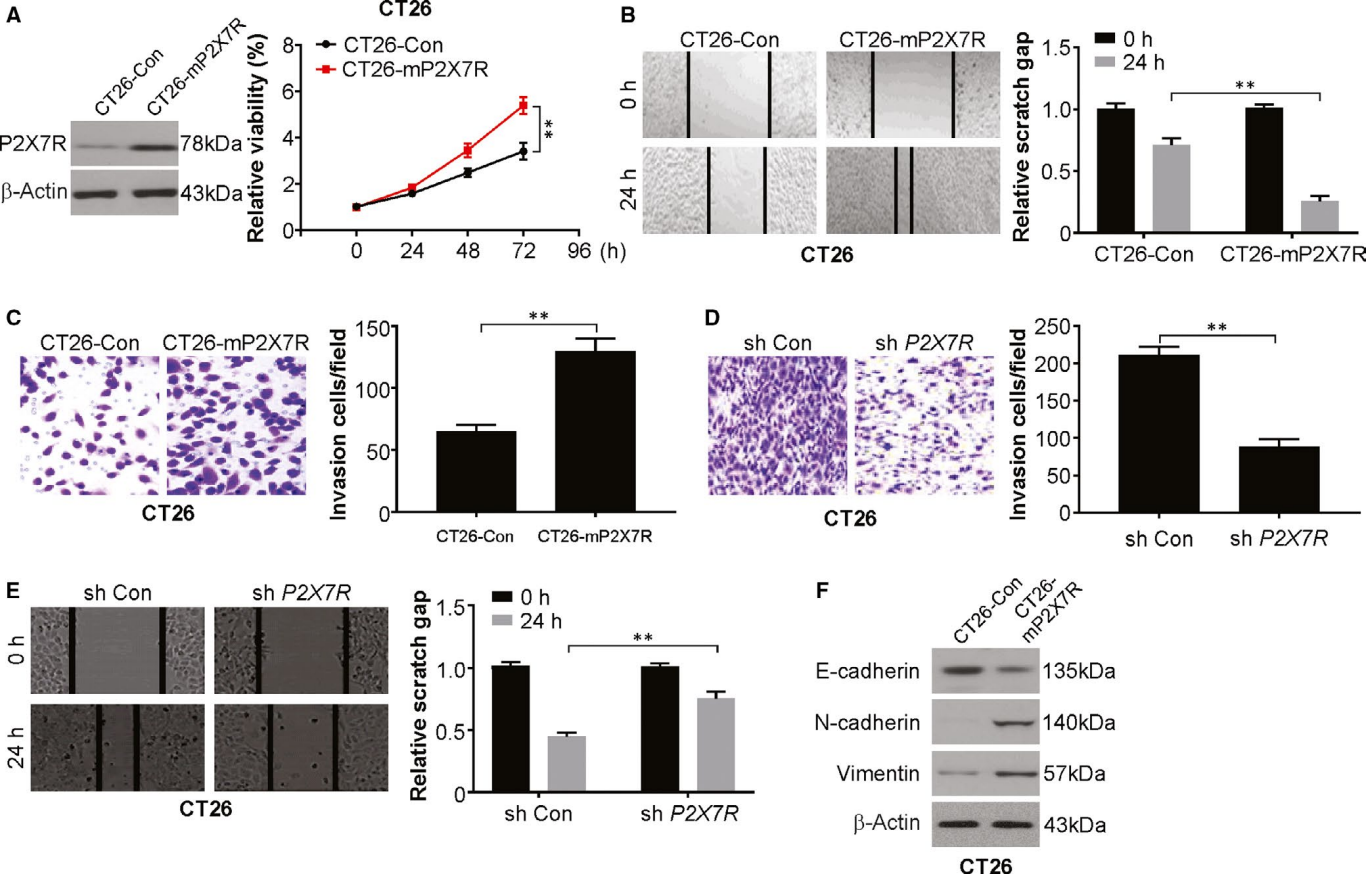
P2X7R promotes proliferation, migration and invasion in colorectal cancer cells. A, Cell proliferation was determined by CCK‐8 assay on the days indicated in CT26‐Con and CT26‐mP2X7R cells. B, Cell migration was determined in CT26‐Con and CT26‐mP2X7R cells using a wound healing assay. The area of the wound was quantified to determine the extent of wound repair. C, 1 × 10^3^ CT26‐Con and CT26‐mP2X7R cells were seeded in transwell chambers. Cell invasion was determined using a Matrigel transwell invasion assay 24 h later. The number of invading cells was quantified after crystal violet staining. D, 5 × 10^3^ CT26‐sh Con and CT26‐sh mP2X7R cells were seeded in transwell chambers. Cell invasion was determined using a Matrigel transwell invasion assay 24 h later. The number of invading cells was quantified after crystal violet staining. E, Cell migration by CT26‐sh Con and CT26‐sh *mP2X7R* cells was determined using a wound healing assay. The area of the wound was quantified to determine the extent of wound repair. F, Western blotting of the indicated proteins in CT26‐Con and CT26‐mP2X7R cells. The results are expressed as the means ± SD of 3 independent experiments. **P < 0.01

To investigate the role of P2X7R in CRC cells, we assessed the in vitro effect of P2X7R overexpression on CT26 cells. Our results indicated that P2X7R promoted the proliferation of CT26 cells (Figure 1A). This was followed by studying the changes in behaviour due to P2X7R overexpression. CT26‐mP2X7R cells migrated at a higher rate compared to that of CT26‐Con cells, as observed in the wound healing assay (Figure 1B). CT26‐mP2X7R cells showed an increased ability to invade compared to that of CT26‐Con cells (Figure 1C). This trend was also displayed by HCT116 and RKO cells in terms of invasion (Figure S1A,B) and migration ability (Figure S1C,D). In contrast, P2X7R knockdown reduced the abilities of CT26 and DLD1 cells to migrate and invade (Figure 1D,E; Figure S1E,H). These observations indicate that the increased abilities of CRC lines to migrate and invade in vitro were due to P2X7R.

A crucial step in tumorigenesis is the epithelial‐mesenchymal transition (EMT), which boosts the ability of epithelial cells to migrate and invade, facilitating metastasis.^31^, ^32^ The involvement of P2X7R in this phenomenon has been documented in many cancers.^33^ This work examined the levels of proteins involved in the EMT by Western blot analysis of CT26‐mP2X7R and CT26‐Con cells. There was a marginal reduction in E‐cadherin and induction of N‐cadherin and vimentin (Figure 1F). shRNA‐mediated blocking of P2X7R resulted in an evident increase in E‐cadherin in CT26 and DLD1 cells (Figure S1I,J). Overall, these observations indicate a lack of likely and vital involvement of the EMT in the response in the cell lines studied to P2X7R overexpression.

### 3.2 P2X7R enhances the growth of CRC tumours in vivo

**FIGURE 2:**
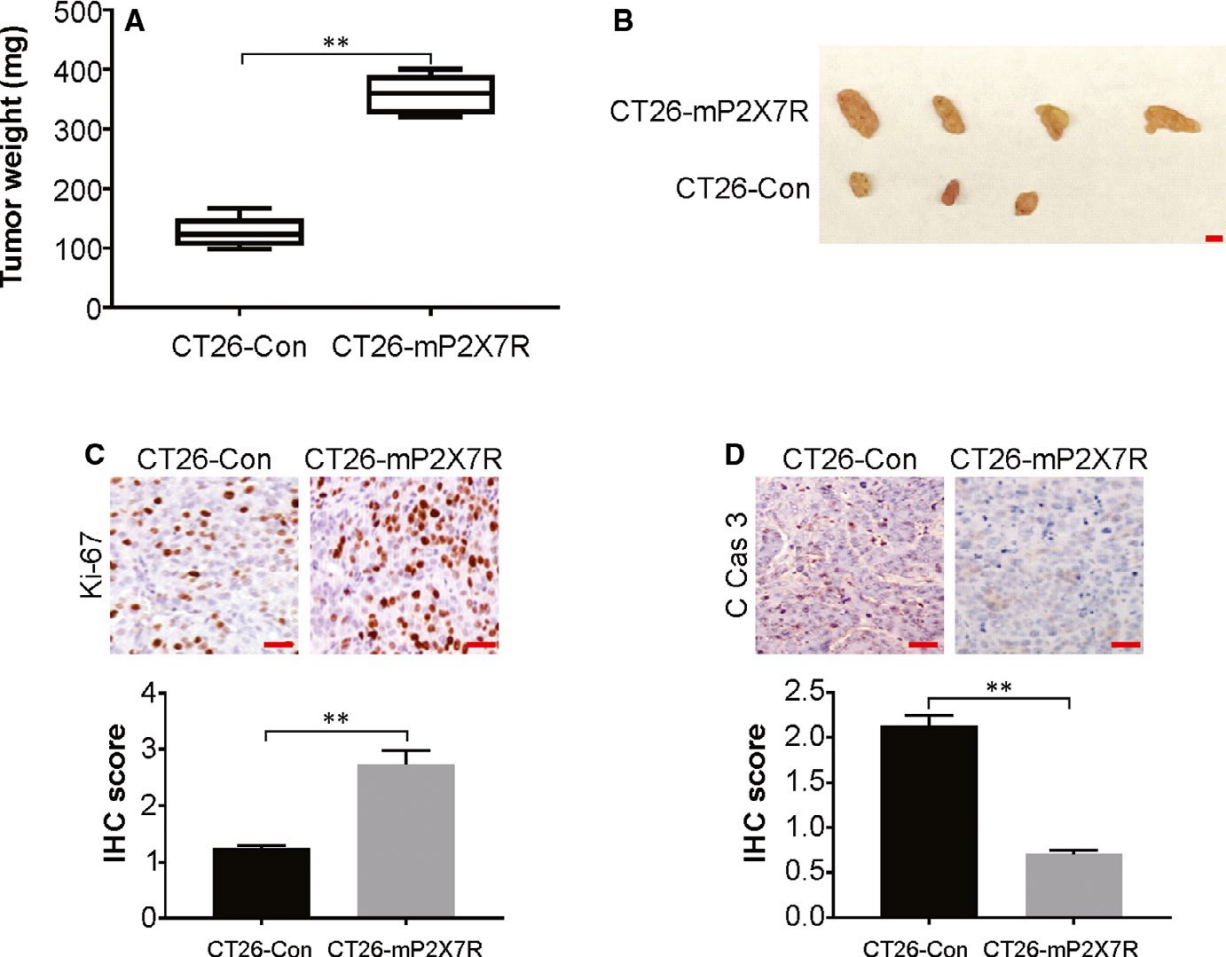
P2X7R overexpression increases colorectal cancer tumour growth in vivo. A, CT26‐Con or CT26‐mP2X7R cells were orthotopically injected into the caecum subserosa of BALB/c mice, and the mice were killed after 6 weeks. The weight of tumours in the mice injected in the caecum with CT26‐Con or isolates of CT26‐mP2X7R cells (*n* = 10). B, Tumours from CT26‐Con and CT26‐mP2X7R mice. Scale bar: 5 mm. C, The expression of Ki67 in tumours from mice injected in the caecum with CT26‐Con and CT26‐mP2X7R cells was analysed by immunohistochemistry. Scale bar: 25 μm. D, The expression of cleaved caspase‐3 in tumours from mice injected in the caecum with CT26‐Con and CT26‐mP2X7R cells was analysed by immunohistochemistry. Scale bar: 25 μm

The involvement of P2X7R overexpression in the advancement of CRC was assessed using a surgical orthotopic mouse model. The formation of primary tumours was observed in 8 of the 10 mice, with an average tumour weight of ~360 mg (Figure 2A,B). This was a stark difference from the tumours formed in 3 of the 10 mice administered CT26‐Con cells, with an average tumour weight of ~110 mg. This shows a conspicuous increase in the growth and weight of tumours due to P2X7R overexpression.

The CT26‐Con or CT26‐mP2X7R tumours that formed in the caecum were assessed for their properties to explore the role of P2X7R in CRC. Immunohistochemistry was performed for Ki67, a proliferation marker, and cleaved caspase‐3, an apoptosis marker, because the above‐mentioned tumour increases may be due to increased proliferation or reduced apoptosis. The number of cells that were positive for Ki67 was higher in CT26‐mP2X7R cells compared to that of CT26‐Con cells, while the expression of cleaved caspase‐3 was lower in CT26‐mP2X7R than CT26‐Con cells (Figure 2C,D), which suggests that P2X7R induces increased proliferation of cells in tumours, as well as reduced apoptosis rates.

### 3.3 P2X7R promotes CRC tumour angiogenesis

**FIGURE 3:**
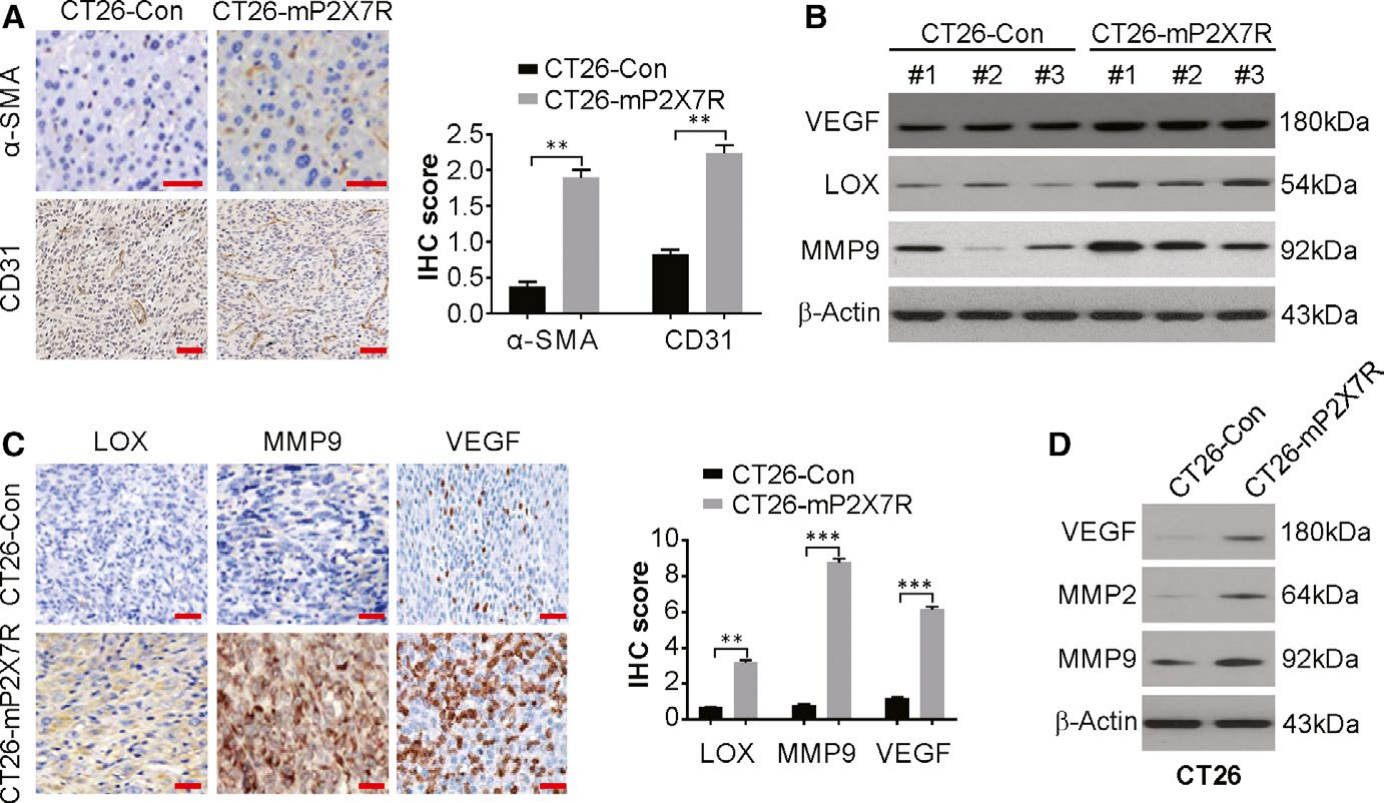
P2X7R overexpression promotes angiogenesis. A, Immunohistochemical staining of CD31 and α‐SMA in CT26‐Con and CT26‐mP2X7R tumours. Scale bar: 25 μm. B, Western blotting of the indicated proteins in sera from CT26‐mP2X7R tumour‐bearing mice and CT26‐Con mice. Albumin was used as a loading control. C, The expression of MMP9, VEGF and LOX in CT26‐Con and CT26‐mP2X7R tumours as analysed by immunohistochemistry. Scale bar: 25 μm. D, Western blotting of the indicated protein in CT26‐Con and CT26‐mP2X7R cells

Tumours expressing high levels of P2X7R and controls displayed the phenotype of a hypercellular solid carcinoma with high mitosis and high‐grade atypia. The occurrence of vascular endothelial cells and pericytes was examined by staining for CD31 and α‐SMA, respectively, in the formed vessels. The staining was more pronounced in the CT26‐mP2X7R tumours compared with that of CT26‐Con tumours (Figure 3A), indicating increased angiogenesis in the tumours due to P2X7R overexpression. This was followed by examining the protein expression of angiogenic and metastatic proteins in the serum of the mice. The protein levels of VEGF, MMP9, MMP2 and LOX increased in the sera of CT26‐P2X7R tumour‐bearing mice compared with those of CT26‐Con mice (Figure 3B). The MMP9, VEGF and LOX levels were higher in CT26‐mP2X7R mice compared to those of CT26‐Con mice, as shown by immunohistochemistry (Figure 3C), which indicates an increase in expression in the serum rather than increased sizes of CT26‐P2X7R compared to that of CT26‐Con mice. Additionally, the in vitro protein levels of MMP9, VEGF and MMP2 were higher in CT26‐mP2X7R cells relative to those of CT26‐Con cells (Figure 3D). This suggests that angiogenesis was stimulated by P2X7R overexpression via the above‐mentioned factors in CRC.

### 3.4 P2X7R increases the features of CSCs in CRC

**FIGURE 4:**
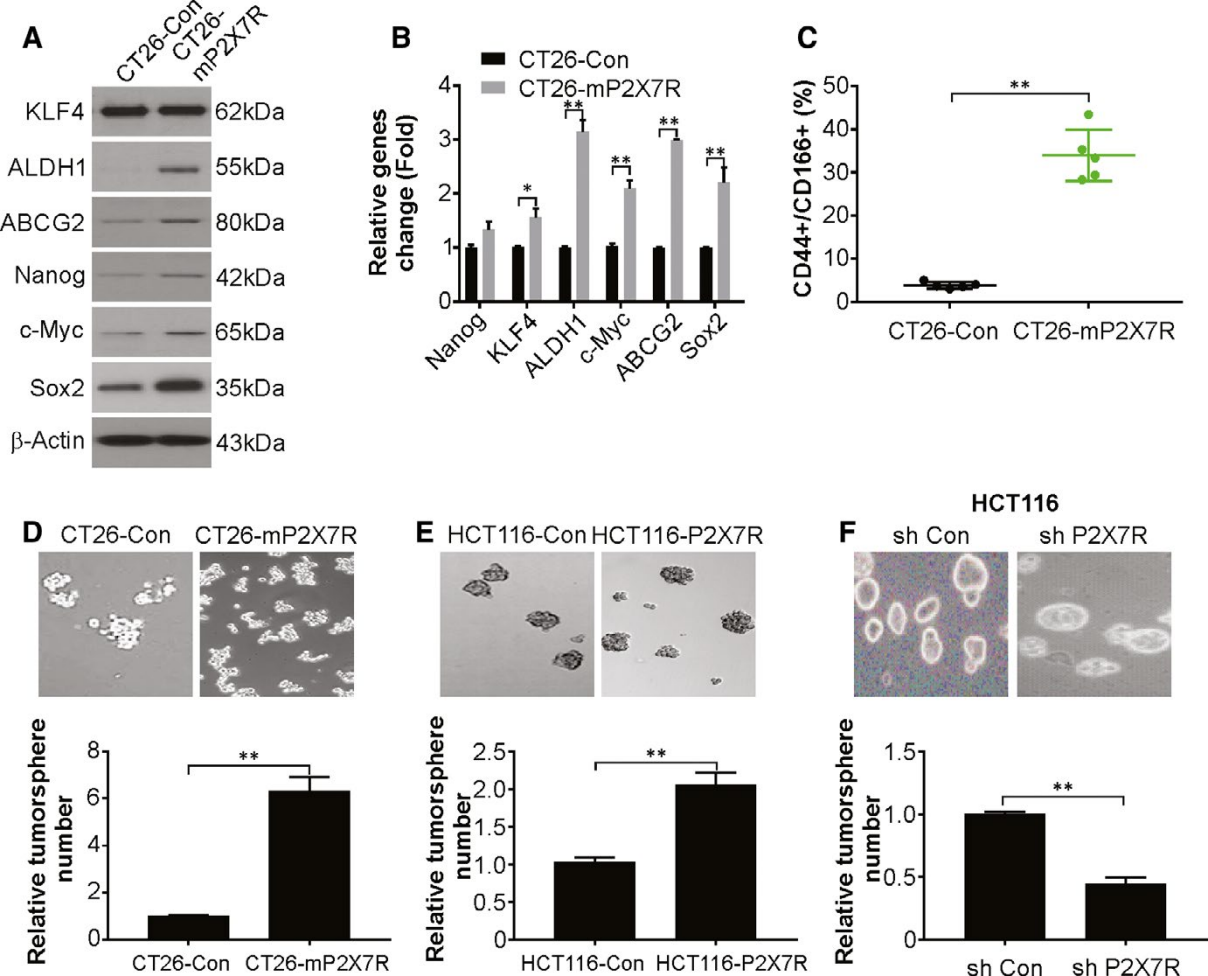
P2X7R overexpression in colorectal cancer (CRC) cells increases CSC characteristics. A, Western blotting of the indicated proteins in CT26‐Con and CT26‐mP2X7R cells. B, mRNA levels of the indicated genes in CT26‐Con and CT26‐mP2X7R cells were analysed by real‐time PCR. C, Analysis by flow cytometry of CD44 and CD166 expression in CT26‐Con or CT26‐mP2X7R cells. D, Tumorsphere formation ability of CT26‐Con or CT26‐mP2X7R cells. E, Tumorsphere formation ability of HCT116‐Con and HCT116‐P2X7R cells. F, Tumorsphere formation ability of HCT116‐sh Con and HCT116‐sh P2X7R cells. The results are expressed as the means ± SD of 3 independent experiments. **P < 0.01

Next, we examined the potential development of CSC features in CRC by P2X7R by examining ALDH1 levels in the tumours and in culture. The ALDH1 levels were considerably higher in the tumours and cultures of CT26‐mP2X7R cells compared to those of CT26‐Con cells (Figure 4A,B). The expression of CD44+ and CD166+ was examined by flow cytometry because these antigens are markers of CSC. There was a conspicuous increase in the number of cells expressing these markers in CT26‐mP2X7R cells compared to that of CT26‐Con cells (Figure 4C). Tumorsphere formation ability was assessed next. CT26‐mP2X7R cells displayed more tumorspheres compared to that of CT26‐Con cells (Figure 4D). A similar study was conducted on human CRC cells with knockdown or overexpression of P2X7R. Overexpression of P2X7R was performed in HCT116 cells because the levels of the receptor are low in this cell line. P2X7R overexpression increased the number of tumorspheres in HCT116 cells (Figure 4E). However, P2X7R knockdown decreased tumorsphere numbers in HCT116 cells (Figure 4F). Hence, the number of cells displaying CSC properties was increased by P2X7R overexpression.

### 3.5 P2X7R boosts TAM recruitment in CT26 tumours

**FIGURE 5:**
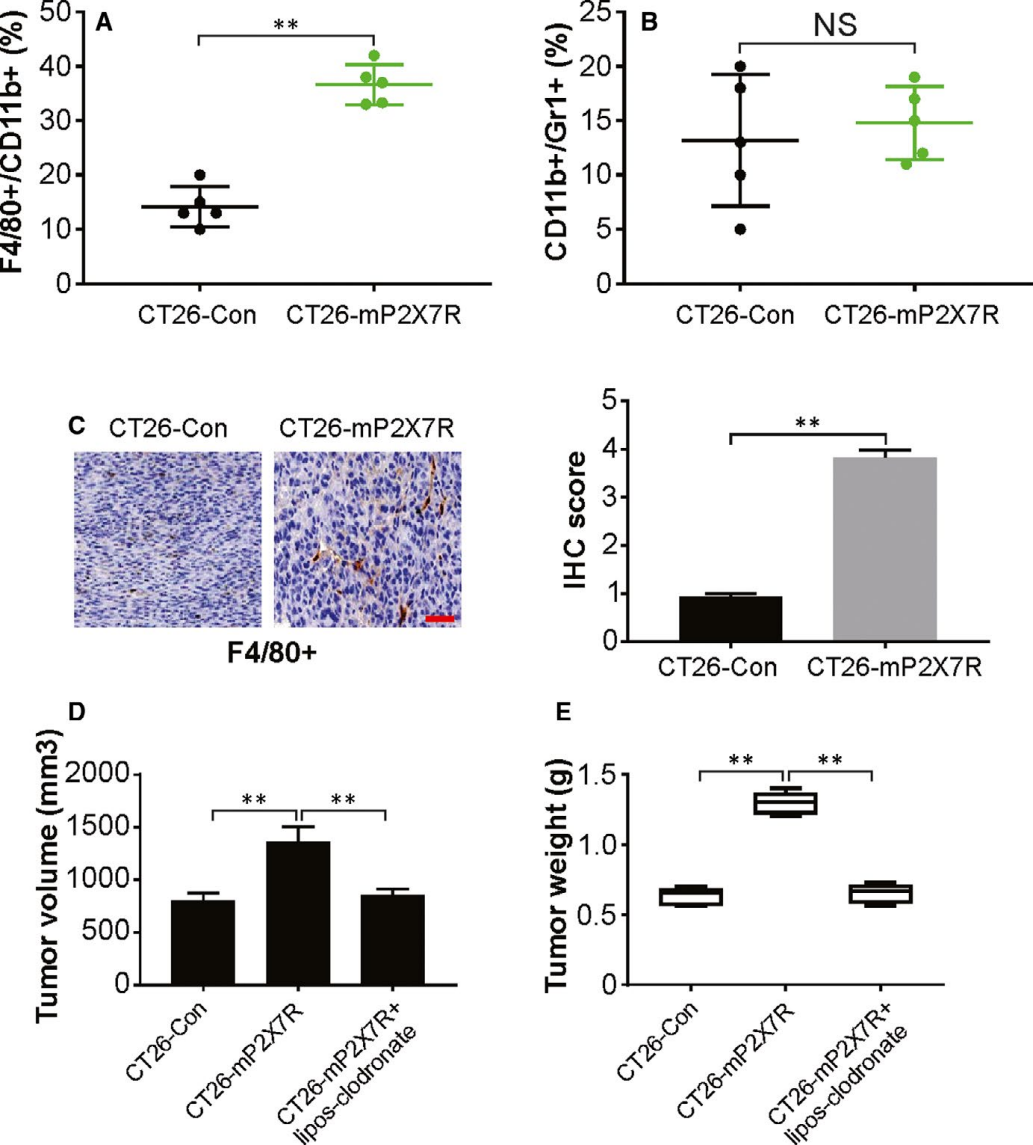
P2X7R promotes tumour‐associated macrophage recruitment. Flow cytometry analysis of the expression of CD11b, F4/80 and Gr1 in CT26‐Con and CT26‐mP2X7R tumours from the caecum. A, The percentage of total cells that were positive for either CD11b+/F4/80+ (a marker for macrophages) or B, CD11b+/Gr1+ (a marker for neutrophils). C, Immunohistochemical staining for the expression of F4/80 in CT26‐Con‐ or CT26‐mP2X7R‐derived tumours. Scale bar: 25 μm. D, Volume of indicated tumours (*n* = 6). E, Tumour weight of indicated tumours. The results are expressed as the means ± SD of 3 independent experiments. **P < 0.01

The infiltration of various types of immune cells, such as TAMs, neutrophils, natural killer (NK) cells and T cells, has been observed in CRC.^34^, ^35^ Flow cytometry was performed to examined the types of infiltrating cells coupled with immunostaining that showed minor alterations in T cells, NK cells, mast cells and B cells (data not shown). CT26‐mP2X7R tumours showed an evident increase in cells expressing macrophage markers (F4/80 and CD11b) compared to that of CT26‐Con tumours, which indicates a boost in infiltration of macrophages due to P2X7R overexpression (Figure 5A). The cell population expressing neutrophil markers (CD11b+ and Gr1+) was reduced in few CT26‐mP2X7R tumours compared with that of the controls but was not significant (Figure 5B). Immunohistochemistry showed that macrophages that were positive for F4/80 formed clusters in CT26‐mP2X7R tumours compared to that of the control CT26‐Con tumours (Figure 5C). Moreover, we used clodronate‐liposome, a macrophage‐depleting agent, to treat the mice. Our findings indicate that clodronate‐liposome attenuated P2X7R‐induced tumour growth (Figure 5D,E). These indicate that P2X7R overexpression results in TAM recruitment and stimulation of angiogenesis in the tumours. This increase in macrophages is likely due to tumour cell recruitment and not systemic inflammation.

### 3.6 P2X7R augments TAM recruitment by increasing cytokines

**FIGURE 6:**
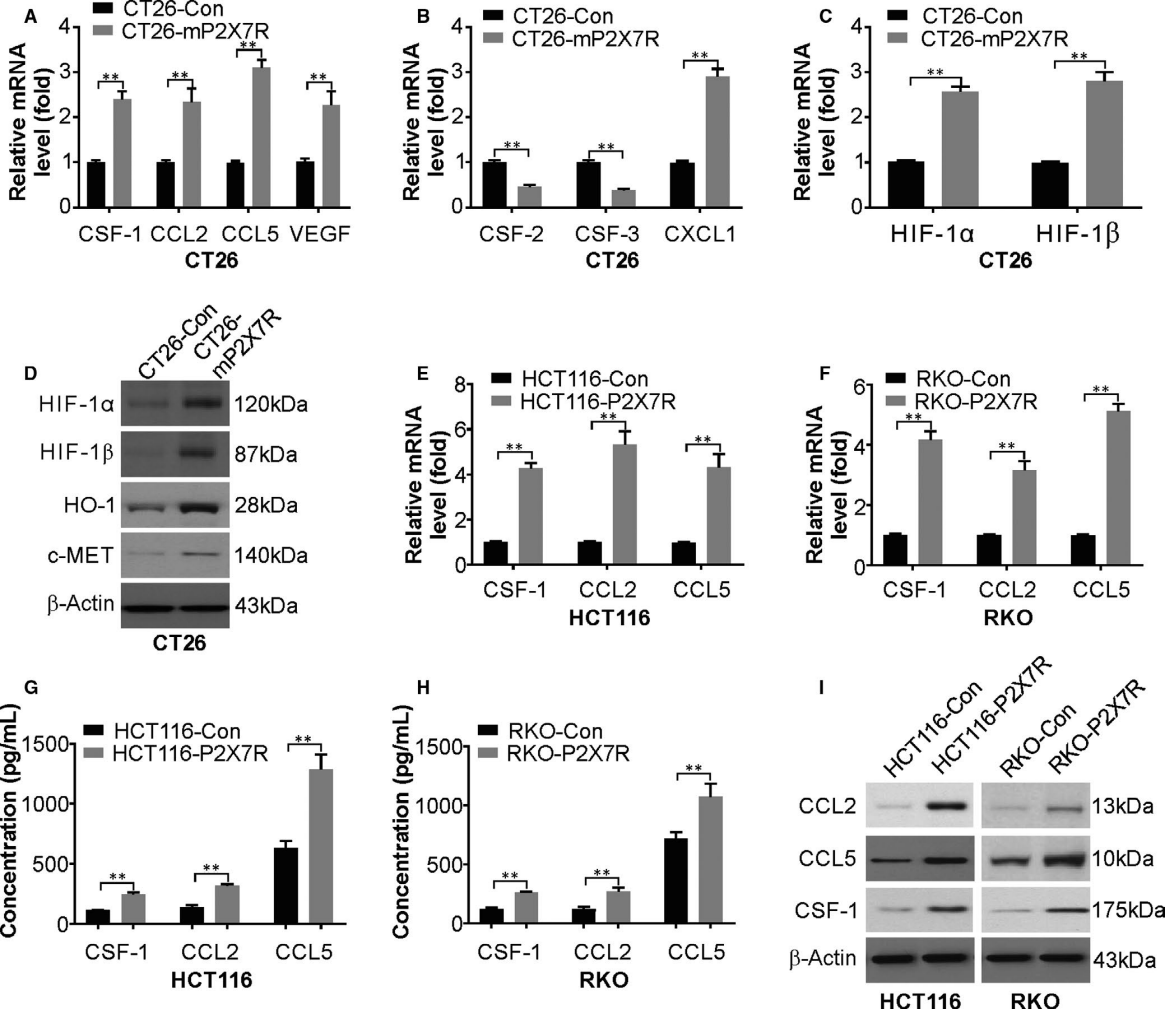
P2X7R overexpression in CT26 cells increases the expression of molecules that induce chemotaxis of TAMs. A, mRNA levels of CSF‐1, CCL2, CCL5 and VEGF in CT26‐Con or CT26‐mP2X7R cells were analysed by real‐time PCR. B, mRNA levels of CSF‐2, CSF‐3 and CXCL1 in CT26‐Con or CT26‐mP2X7R cells were analysed by real‐time PCR. C, mRNA levels of HIF‐1α and HIF‐1β in CT26‐Con or CT26‐mP2X7R cells were analysed by real‐time PCR. D, Protein levels of HIF‐1α, HIF‐1β, VEGFR and MMP2 in CT26‐Con or CT26‐mP2X7R cells were analysed by Western blotting. E, mRNA levels of CCL2, CCL5 and CSF‐1 in HCT116‐Con or HCT116‐mP2X7R cells were analysed by real‐time PCR. F, mRNA levels of CCL2, CCL5 and CSF‐1 in RKO‐Con or RKO‐mP2X7R cells were analysed by real‐time PCR. G, Levels of CCL2, CCL5 and CSF‐1 in the medium of HCT116‐Con or HCT116‐mP2X7R cells were analysed by ELISA. H, Levels of CCL2, CCL5 and CSF‐1 in the medium of RKO‐Con or RKO‐mP2X7R cells were analysed by ELISA. I, Western blotting of CCL2, CCL5 and CSF‐1 in HCT116‐Con, HCT116‐P2X7R, RKO‐Con and RKO‐P2X7R cells. The results are expressed as the means ± SD of 3 independent experiments. **P < 0.01

Through the secretion of growth factors, chemokines and cytokines, TAMs exert pro‐tumour functions by enhancing angiogenesis and tissue remodelling rather than cytotoxic activity.^36^ The recruitment of TAMs to CRC tumours involves CCL2, CCL5, VEGF and CSF‐1.^37^, ^38^ The mRNA levels of these molecules were higher in CT26‐mP2X7R cells compared to those of CT26‐Con cells (Figure 6A). The mRNA expression of cytokines involved in neutrophil recruitment to cancer sites, such as colony‐stimulating factor‐2 (CSF‐2), CSF‐3 and chemokine (C‐X‐C motif) ligand 1 (CXCL1), was determined, and CSF‐2 and CSF‐3 were reduced and CXCL1 expression was increased in CT26‐mP2X7R cells (Figure 6B). The mRNA levels of HIF‐1α and HIF‐1β were assessed in CT26‐Con and CT26‐mP2X7R cells because TAM recruitment was observed in tumours and hypoxic sites. The mRNA and protein levels of both of these genes were increased in CT26‐P2X7R cells (Figure 6C,D). Expression of cytokines associated with macrophage recruitment was also assessed in human cell lines. The mRNA (Figure 6E,F) and protein (Figure 6G‐I) levels of CCL2, CSF‐1 and CCL5 were higher in HCT116 and RKO cells overexpressing P2X7R compared with those of the controls. Thus, these data suggest an increase in cytokines that are involved in the infiltration of macrophages in tumours, which manifests as tumorigenesis.

### 3.7 Augmented and prolonged NF‐кB signalling by P2X7R

**FIGURE 7:**
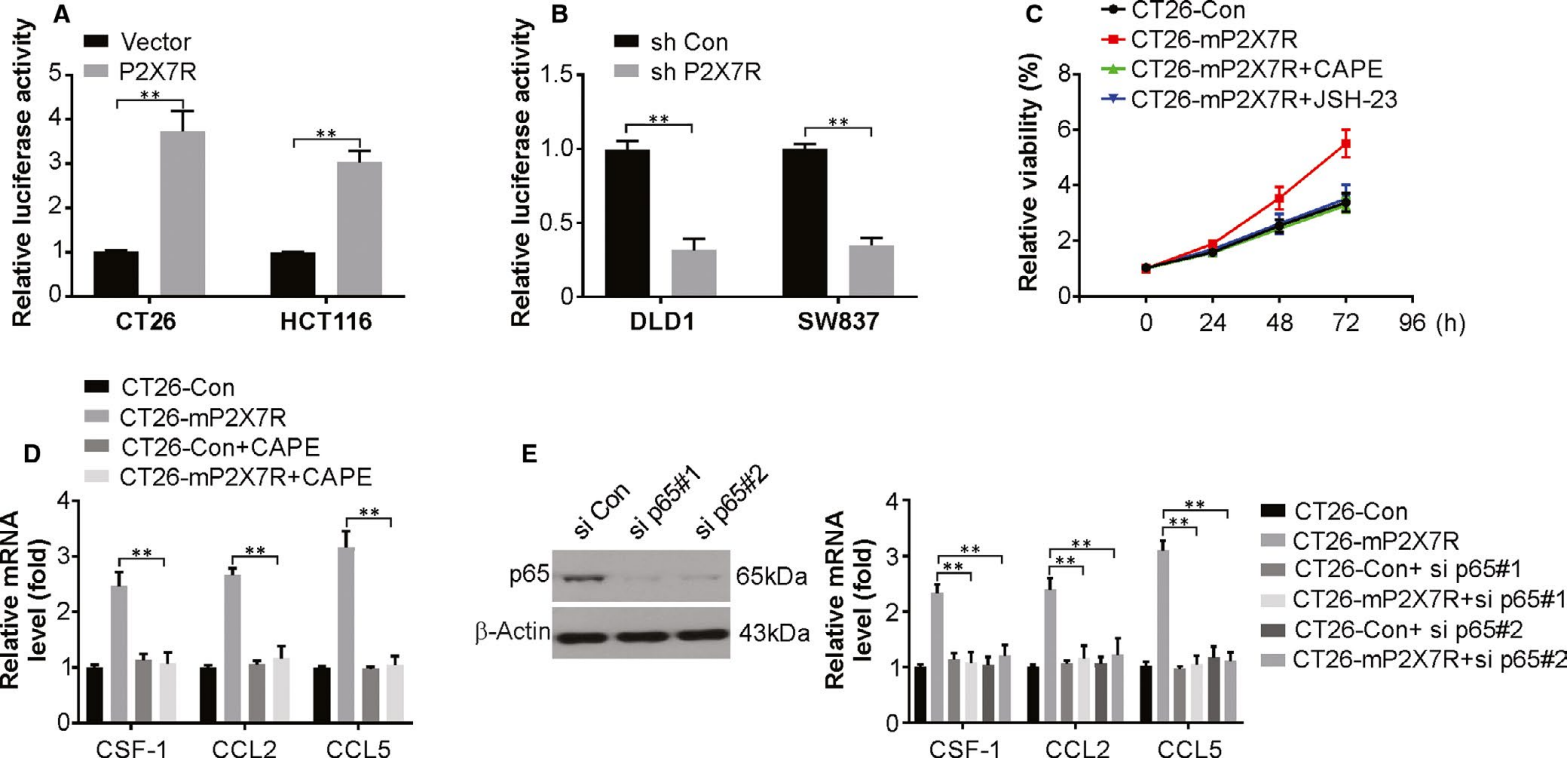
The NF‐κB signalling pathway is responsible for P2X7R‐induced cytokine expression. A, Effects of P2X7R overexpression on the transcriptional activity of NF‐κB in CT26 and HCT116 cells. B, Effects of P2X7R knockdown on the transcriptional activity of NF‐κB in DLD1 and SW837 cells. C, Effects of CAPE (100 nmol/L) and JSH‐23 (200 nmol/L) on cell viability of cells transfected with P2X7R. D, Relative mRNA levels of the indicated cytokines in CT26 cells transfected with P2X7R with or without CAPE (100 nmol/L) treatment. E, Relative mRNA levels of the indicated cytokines in CT26 cells transfected with P2X7R with or without si *p65* cotransfection. The results are expressed as the means ± SD of 3 independent experiments. **P < 0.01

The mechanism of P2X7R in tumorigenesis was assessed by studying many pathways involved in cancer using luciferase reporter assays. The pathways studied included NF‐κB (nuclear factor‐κB), p53, Wnt/β‐catenin (TOP flash/FOP flash), TGF‐β, p21, STAT3 (APRE), p38 and STAT5 (LHRE) (data not shown). The expression of ectopic P2X7R distinctly increased the reporter expression of NF‐кB luciferase in CT26 and HCT116 cells (Figure 7A). This signalling was inhibited by P2X7R knockdown in DLD1 and SW837 cells (Figure 7B). Next, CAPE and JSH‐23, specific inhibitors of NF‐κB, were used to treat cells that expressed or lacked expression of ectopic P2X7R. The proliferation of CT26 cells was markedly lowered by treatment with the inhibitors, which suggests that the NF‐кB pathway is activated in CRC tumorigenesis by P2X7R (Figure 7C). Furthermore, CAPE treatment or p65 knockdown blocked P2X7R overexpression‐induced CSF‐1 and CCL2/5 induction (Figure 7D,E). The above data indicate that the NF‐кB signalling pathway mediates P2X7R‐induced generation of CSF‐1 and CCL2/5, TAM recruitment, and tumour progression.

## 4 DISCUSSION

In the current study, our findings demonstrated P2X7R overexpression is relative to tumour growth, migration and invasion in vitro. Our results also indicated that P2X7R overexpression augmented tumorigenesis and angiogenesis in the mouse CT26 cell line in vivo. Moreover, P2X7R overexpression results in the CSC properties. In addition, P2X7R promotes TAM recruitment, which is required for P2X7R‐mediated tumour progression. The NF‐κB signalling pathway was found to be activated and may play a role in the function of P2X7R‐induced cytokines induction as well as tumour growth in CRC cells. A vital feature behind the aggressiveness of CRC is the spread of cells due to migration and invasion. P2X7R overexpression was found to boost these abilities in vitro.

The EMT is a vital step in metastasis that is manifested as a deficit in E‐cadherin.^39^ This study reported a conspicuous reduction in E‐cadherin in CRC cells and an induction in N‐cadherin and vimentin in CRC cells with P2X7R overexpression. P2X7R knockdown resulted in an increase in E‐cadherin, as well as N‐cadherin and vimentin reduction in CRC cells. These results suggest the possibility of EMT involvement in the ability of CRC to migrate and invade in certain instances, with the degree of transition. P2X7R overexpression also increased VEGF, LOX and MMP9, which increased blood vessel formation, with α‐SMA and CD31 positive cells increased, suggesting a mechanism for P2X7R in CRC tumorigenesis.

The formation of new blood vessels supports the metabolic needs of tumours as well as allowing for the escape of cells to spread the disease.^40^ Tumour cells were implanted to construct a murine model that showed that the higher levels of P2X7R caused changes in the tumour stroma via TAM recruitment and angiogenesis, as well as increased CSC features. The tumour microenvironment is critical in the process of metastasis.^41^ As previously study, metastasis involves M2 but not M1 macrophages that support tumour formation.^42^ This stage prior to metastasis involves multiple factors, including cytokines and immune cells, such as neutrophils and regulatory/suppressor cells.^43^ The formation of the pre‐metastatic niche involves the secretion of factors by the tumour to stimulate resident macrophages and recruit macrophages/monocytes.^44^ However, the involvement of TAMs as safe havens for tumour cells, especially in distant organs, has recently received more widespread research attention. The tumour microenvironment involves crucial stromal cells that undergo recruitment by cancer cells to boost the advancement and metastasis.^45^ Angiogenesis is initiated by the secretion of VEGF, a major factor that causes the induction and proliferation of endothelial cells.^46^

The involvement of macrophages or TAMs in tumorigenesis is being unravelled.^47^ In the first stages, a proinflammatory environment is maintained by the macrophages, while in later steps, angiogenesis is favoured with an augmentation of the ability of tumour cells to migrate and invade and control antitumour immunity.^48^ CRC and other cancer types show high levels of CSF‐1, a vital macrophage regulator that is linked to poor prognosis.^49^ The expression of transcription factors regulating angiogenesis, such as HIF‐1α, is constitutively observed in macrophages that are located in hypoxic tumour sites.^50^ MMP9 synthesized by macrophages upon cues from tumour cells allows for invasion by disruption of the extracellular matrix.^51^ The present work reports infiltration of TAMs by altering the expression of several chemokines, such as CSF‐1, CCL2 and CCL5, in a murine model. These TAMs produce molecules that favour tumour biology, such as MMP9 and VEGF that allow for angiogenesis and metastasis.

In summary, the present work indicates that P2X7R overexpression promotes cancer aggressiveness and correlates with macrophage infiltration in CRC and provides new insight into the roles of P2X7R in immune modulation. These findings point towards the P2X7R/NF‐κB/CSF‐1/CCL2/5/macrophage axis as potential therapeutic targets in CRC.

## CONFLICTS OF INTEREST

The authors declare that they have no conflicts of interest.

## AUTHOR CONTRIBUTION

**Chunhui Yang:** Data curation (equal); investigation (equal). **Shuang Shi:** Data curation (equal); investigation (equal). **Ying Su:** Data curation (equal); investigation (equal). **Liangjun Li:** Data curation (equal); investigation (equal); project administration (equal). **Jingshan Tong:** Project administration (equal).

## Supporting information

Fig S1

## Data Availability

The data that support the findings of this study are available from the corresponding author upon reasonable request.
