# Supplementary material for: P2X7R promotes angiogenesis and tumour‐associated macrophage recruitment by regulating the NF‐κB signalling pathway in colorectal cancer cells
Source: J Cell Mol Med. 2020 Jul 31;24(18):10830–41. doi: 10.1111/jcmm.15708 (PMC7521273; doi:10.1111/jcmm.15708)
Supplement: Supplementary file 1 — Fig S1 [file JCMM-24-10830-s001.docx]

**P2X7R promotes angiogenesis and tumor-associated macrophage recruitment by regulating the NF-κB signaling pathway in colorectal cancer cells**

Chunhui Yang^1^, Shuang Shi^1^, Ying Su^1^, Jing-Shan Tong^2*^, Liangjun Li^1*^

1. Department of Clinical Laboratory, the Second Affiliated Hospital of Dalian Medical University, Dalian, Liaoning, China
2. Department of Pharmacology and Chemical Biology, University of Pittsburgh School of Medicine, Pittsburgh, PA, USA

Chunhui Yang, Shuang Shi and Ying Su contributed equally to this work.

Correspondence authors:

Liangjun Li, Department of Clinical Laboratory, the Second Affiliated Hospital of Dalian Medical University, No. 467 Zhongshan Road, Shahekou District, Dalian, Liaoning 116027, China. Email: [dy2yliliangjun@163.com](mailto:dy2yliliangjun@163.com);

Jing-Shan Tong, UPMC Hillman Cancer Center, 5117 Centre Ave, Pittsburgh, PA 15213, USA. Email: [tongjingshan@gmail.com](mailto:tongjingshan@gmail.com)

**Running title:** P2X7R regulates angiogenesis and TAMs


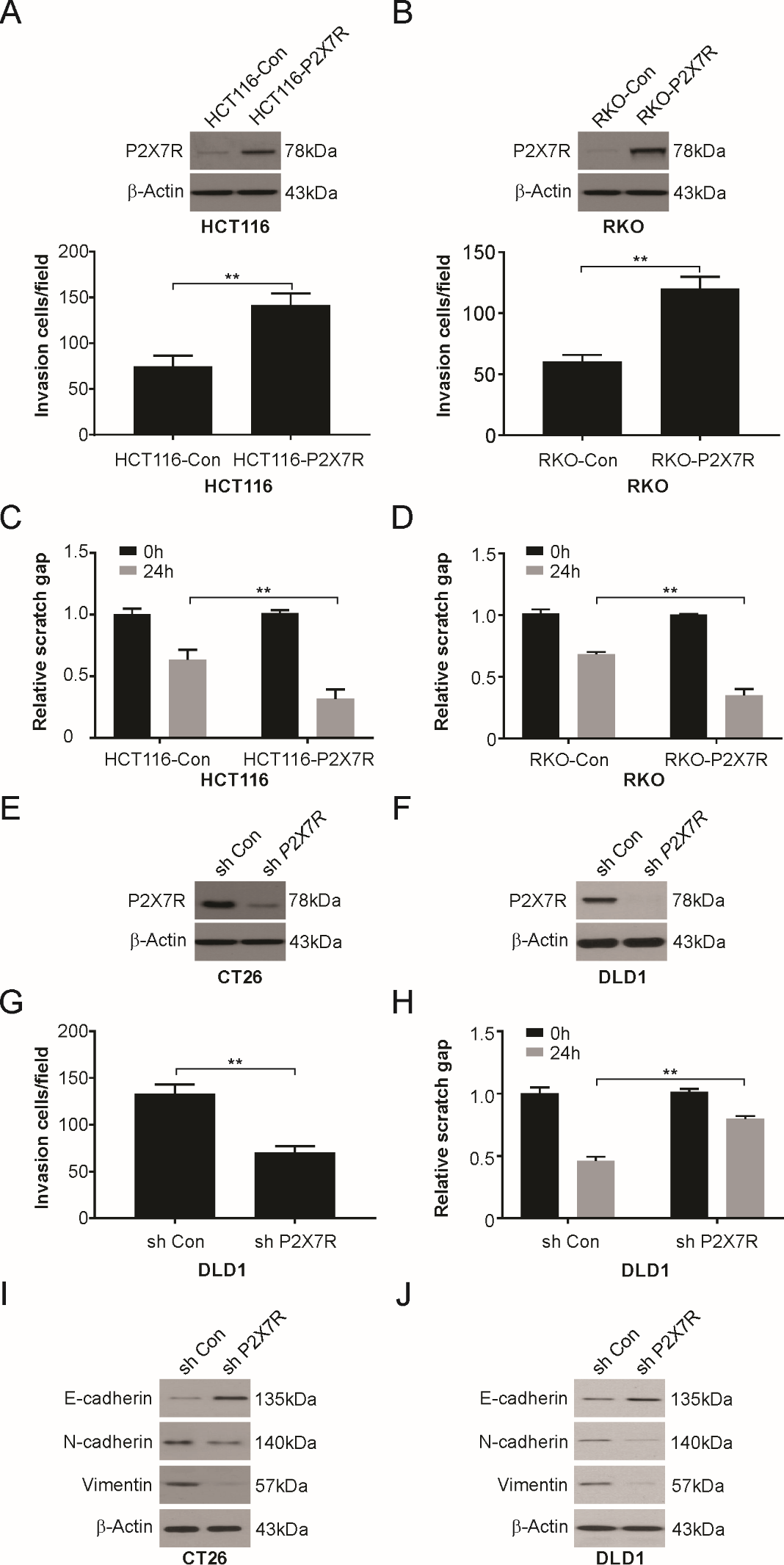


**Figure S1. (A)** Cell invasion of HCT116-Con and HCT116-P2X7R cells was determined by Matrigel Transwell invasion assay. **(B)** Cell invasion of RKO-Con and RKO-P2X7R cells was determined by Matrigel Transwell invasion assay. **(C)** Cell migration of HCT116-Con and HCT116-P2X7R cells was determined by a wound healing assay. The area of the wound as quantified to determine the extent of wound repair. **(D)** Cell migration of RKO-Con and RKO-P2X7R cells was determined by a wound healing assay. The area of the wound was quantified to determine the extent of wound repair. **(E)** P2X7R level in CT26-sh Con and CT26-sh P2X7R cells was analyzed by Western blotting. **(F)** P2X7R level in DLD1-sh Con and DLD1-sh P2X7R cells was analyzed by Western blotting. **(G)** Cell invasion of DLD1-sh Con and DLD1-sh P2X7R cells was determined by Matrigel Transwell invasion assay. **(H)** Cell migration of DLD1-sh Con and DLD1-sh P2X7R cells was determined by a wound healing assay. The area of the wound was quantified to determine the extent of wound repair. **(I)** Western blotting of the indicated proteins in CT26-sh Con and CT26-sh mP2X7R cells. **(J)** Western blotting of the indicated proteins in DLD1-sh Con and DLD1-sh mP2X7R cells. The results are expressed as the means ± SD of 3 independent experiments. **, *P* <0.01.
